# Global Antimicrobial Resistance and Use Surveillance System on the African continent: Early implementation 2017–2019

**DOI:** 10.4102/ajlm.v11i1.1594

**Published:** 2022-08-31

**Authors:** Barbara Tornimbene, Sergey Eremin, Reuben Abednego, Elamin O. Abualas, Ilhem Boutiba, Abiodun Egwuenu, Walter Fuller, Laetitia Gahimbare, Susan Githii, Watipaso Kasambara, Chileshe Lukwesa-Musyani, Fidy A. Miamina, Sekesai Mtapuri-Zinyowera, Grace Najjuka, Olga Perovic, Bassem Zayed, Yahaya A. Ahmed, Maha T. Ismail, Carmem L. Pessoa da Silva

**Affiliations:** 1 AMR Division, Surveillance, Prevention and Control Department, World Health Organization, Geneva, Switzerland; 2 National Health Laboratory Quality Assurance and Training Centre (NHLQATC), Tanzania, Dar es Salaam, United Republic of Tanzania; 3 National Public Health Laboratory, Federal Ministry of Health, Khartoum, Sudan; 4 Faculty of Medicine, University of Tunis El Manar, Tunis, Tunisia; 5 Nigeria Center for Disease Control, Abuja, Nigeria; 6 Antimicrobial Resistance (AMR) World Health Organization, Regional Office for Africa, Brazzaville, Congo; 7 National Microbiology Reference Lab, National Public Health Laboratories, Nairobi, Kenya; 8 Ministry of Health, Lilongwe, Malawi; 9 Lusaka District Laboratory, University of Zambia, Lusaka, Zambia; 10 Department of Health Watch, Epidemiological Surveillance and Response (DVSSER), Antananarivo, Madagascar; 11 National Microbiology Reference Laboratory, Zimbabwe, Harare, Zimbabwe; 12 Department of Microbiology, Joint Clinical Research Centre (JCRC), Kampala, Uganda; 13 Centre for Healthcare-Associated Infections, Antimicrobial Resistance and Mycoses (CHARM), Johannesburg, South Africa; 14 World Health Organization, Regional Office for East Mediterranean, Cairo, Egypt; 15 World Health Organization, Regional Office for Africa, Brazzaville, Congo

**Keywords:** AMR, surveillance, Africa, implementation, WHO

## Abstract

**Background:**

Antimicrobial resistance (AMR) is becoming a critical public health issue globally. The World Health Organization launched the Global Antimicrobial Resistance and Use Surveillance System (GLASS) to support the strengthening of the AMR evidence base.

**Objective:**

The article describes the evolution of national AMR surveillance systems and AMR data reporting of countries in the African continent between 2017 and 2019, and the constraints, perceived impact and value of the participation in GLASS.

**Methods:**

Data on implementation of national surveillance systems and AMR rates were submitted to GLASS between 2017 and 2019 and summarised though descriptive statistics. The information on constraints and perceived impact and value in GLASS participation was collected though a set of questionnaires.

**Results:**

Between 2017 and 2019, Egypt, Ethiopia, Madagascar, Malawi, Mali, Mozambique, Nigeria, South Africa, Sudan, Tunisia, Uganda and Zambia submitted data to GLASS. The main constraints listed are linked to scarce laboratory capacity and capability, limited staffing, budget issues, and data management. Moreover, while the data are not yet nationally representative, high resistance rates were reported to commonly-used antibiotics, as the emerging resistance to last treatment options.

**Conclusion:**

Despite the limitations, more and more countries in the African continent are working towards reaching a status that will enable them to report AMR data in a complete and systematic manner. Future improvements involve the expansion of routine surveillance capacity for several countries and the implementation of surveys that allow to effectively define the magnitude of AMR in the continent.

## Introduction

Antimicrobial resistance (AMR) is defined as ‘the presence of resistance to antimicrobial medicines in infectious agents such as bacteria, viruses, fungi and parasites’^1^. This resistance can be inherent or acquired by the inappropriate use of medicines. Recent studies position AMR as a leading cause of death around the world, with the highest burdens in low-resource settings.^2^

Africa remains the continent most afflicted by infectious diseases and AMR can dramatically hamper treatment effectiveness and greatly amplify disease burden and its complications.^3^ A 2017 systematic review from Tadesse et al.,^4^ documenting the status of AMR in Africa, found that ‘recent AMR data was not available for more than 40% of countries’. In countries for which data were available, ‘the level of resistance to commonly prescribed antibiotics was significant’, and that the ‘quality of microbiological data is of serious concern’.^3^

A second systematic review, also published in 2017 and targeting West Africa, found AMR to be common in this subregion. It particularly occurred in hospitalised patients with bloodstream infections (BSI), and both outpatient and hospitalised patients with urinary tract infection (UTI).^5^ Two reviews of AMR in sub-Saharan Africa and one review in East Africa also revealed ‘a high prevalence of AMR to commonly-used antibiotics in clinical bacterial isolates’.^6,7^ The studies also flagged the flaws in available data and the challenges faced in low- and lower middle-income countries when implementing AMR surveillance.

In 2015, the World Health Organization (WHO) launched the Global Antimicrobial Resistance and Use Surveillance System (GLASS) to support strengthening of the AMR evidence base. As stated in the GLASS Report 2020, GLASS encourages countries to move to surveillance approaches based on systems that includes epidemiological, clinical, and population-level data, rather than only on laboratory data. In addition to data collection, GLASS promotes strengthening of national AMR surveillance systems to ensure that reliable and representative information is produced. It is supported by WHO Collaborating Centres Network, involving strong commitment from participating countries and close collaborations with WHO Headquarter, Regional and Country Offices.^8^

During the early implementation phase (2015–2019), GLASS focused on collecting information on the status of existing or newly developed national AMR surveillance systems and to provide a standardised approach to the collection of AMR rates for selected bacteria causing generic infections in humans. The first data call was opened in May 2017, and it recurs every year in the same period, between 1 May and 31 August.

This article aims to describe the evolution of national AMR surveillance systems and AMR data reporting capacity of African countries participating in GLASS for the first three annual GLASS data calls (2017–2019), together with a summary of reported AMR rates for selected indicators. The article also describes, for a subset of African countries, the constraints, perceived impact, and value linked to reporting data to GLASS.

## Methods

### Ethical considerations

Ethics approval was not required for this study. Each country has its own ethical approval for AMR surveillance and in many cases routine surveillance does not require ethical clearance.

### Data sources

The GLASS database is the source of information of countries participation and reporting, implementation of the national surveillance system, and AMR rates. The data were submitted by counties during three data calls, between 2017 and 2019. The information on constraints and perceived impact and value in GLASS participation was collected though a set of questions sent via email to countries’ AMR National Focal Points (NFPs) and WHO Regional Offices’ staff in charge of AMR activities.

### Information gathered thorough Global Antimicrobial Resistance and Use Surveillance System data calls

The GLASS AMR data call is open yearly between May and August, and countries submit the information on the implementation of the national AMR surveillance systems for the data call year, and AMR rates for the previous year.

#### Information on the implementation of the national surveillance system

In this article, key indicators on the implementation of the national surveillance system are summarised and presented to reflect changes during three data calls (2017–2019).

As stated in the GLASS Report 2020,^7^ GLASS collects information on the implementation of national AMR surveillance systems through a standardised questionnaire filled in every year by the NFP.

A set of indicators is then used to measure the development and strengthening of national AMR surveillance:^7^

- The establishment of a National Coordinating Centre and the National Reference Laboratory. These two bodies are in charge of data management and capacity building, and are key to the coordination of the national systems.
- Number of surveillance sites and local laboratories performing Antimicrobial Susceptibility Testing (AST) that report AMR data to GLASS. This information allows a better understanding of the structure and capacity of the national surveillance system structure. Surveillance sites can be hospitals, clinics, or in-and outpatient community healthcare facilities with access to relevant epidemiological and laboratory support and information.
- Provision of External Quality Assessment. This allows a better understanding of the diagnostic capacity of the surveillance system, by checking the provision of External Quality Assessment to the National Reference Laboratory and clinical local laboratories, and the use of international standards for diagnostics and AST.

#### Antimicrobial resistance data

As stated in the GLASS Reports, GLASS requires AMR data to be collected through a surveillance system that gathers results from AST for common human bacterial pathogens in four infection sites, specifically BSIs caused by *Acinetobacter* spp., *E. coli, K. pneumoniae, Salmonella* spp., *S. aureus* and *S. pneumoniae*, UTIs caused by *E. coli and K. pneumonia*, gastrointestinal infections caused by *Salmonella* spp. and *Shigella* spp., and genital infections caused by *N. gonorrhoeae*. Data are generated by the collation of results from specimens that are routinely sent to laboratories for clinical testing and includes blood, urine, stool and cervical and urethral samples. The rationale for selection of these particular specimens is that the growth of a pathogen is a proxy of infection in the associated anatomical sites. The target population under surveillance is the national population of patients seeking care in HCFs. Data to be collected includes: ‘numbers of patients with susceptible, non-susceptible, intermediate, and resistant isolates, as well as numbers of isolates with unknown susceptibility.^8,9,10^ Two types of unknown results are recorded. The first, ‘unknown_no_AST’, is the number of isolates with AST results that were not reported (or not performed) for a specific antibiotic. The second, ‘unknown_no_breakpoints’, is the number of isolates for which AST was performed but which had no interpretation of results available for a specific antibiotic. Additionally, countries are invited to report patients’ microbiological results (bacterial isolation and identification, AST), as well as demographic and epidemiological variables such as age, gender, and origin of infection in tested patients, in aggregated format.^8^

The distribution of infections and bacteria analysed and submitted to GLASS by countries during the three data calls (2017–2019) is summarised by year and shown in table format.

**Antimicrobial resistance rates:** Proportions of patients with resistant infections reported by countries during three data calls are presented for:

- Bloodstream infections caused by *Escherichia coli* and *Klebsiella pneumoniae* resistant to carbapenems and third-generation cephalosporins (3GC), and BSIs caused by methicillin-resistant *Staphylococcus aureus* (MRSA).
- Urinary tract infection caused by *E. coli* and *K. pneumoniae* resistant to carbapenems and ciprofloxacin.

As aligned to the method outlines in the GLASS Report 2016–2017, ‘rates are shown only if [*countries reported*] results for > 10 patients, and for pathogen–antibiotic combinations with > 10 AST results and < 30% unknown AST results’^11^.

Box-and-whisker plots are used to summarise the reported median rate of resistance for specific specimen–pathogen–antibiotic combinations. The plots portray the distribution of the submitted data, outliers, and the median. The box within the chart displays where around 50% of the data points fall and it contains the lower quartile, the upper quartile, and the median in the centre. The median is the value separating the higher half from the lower half of the results.

### Information gathered through countries’ and WHO Regional Offices’ feedback

After the end of the first GLASS data call in August 2017 and the last data call in August 2019, enrolled African countries, the WHO Regional Office for Africa and the WHO Regional Office for the Eastern Mediterranean were asked to provide feedback to a set of questions covering three themes:

- Constraints: the difficulties encountered to participate in GLASS data calls.
- Impact: the positive impact that the three data calls might have had to foster data generation.
- Value: the added value of participating in GLASS.

The qualitative data obtained from countries’ and Regional Offices’ feedback is summarised in the article using a set of codes identified for each theme and shown in pie chart format. Coding was done by identifying a passage in the text, searching, and identifying concepts, and finding relationships between them.

#### Data analysis

Data on information on the implementation of the national surveillance system were exported from the GLASS information technology platform into Microsoft Excel (Microsoft corporation, California, United States), and bar charts were used to visualise the proportion of each variable outcome for all reporting countries in order to interpret the data.

Antimicrobial resistance data were exported from the GLASS information technology platform and validated and analysed using STATA (StataCorp, College Station, Texas, United States) software. For each country, the number of patients with confirmed bacterial infection was calculated by collapsing all the laboratory results (susceptible, non-susceptible, intermediate, and resistant isolates, as well as numbers of isolates with unknown susceptibility) for a specific pathogen and choosing the antibiotic with the highest number of results reported. The number of patients with AST results by pathogen is calculated in the same way, but unknown susceptibility results are not included. The proportion of patients with resistant infection for a specific indicator (see ‘Antimicrobial resistance rates’ mentioned earlier) is then calculated for each country using the following formula:

$$\begin{array}{l} (\text{Number of patients, per specimen type, with infection by}\\ \text{pathogen}\times \text{resistant to antibiotic y under surveillance / Total}\\ \text{number of patients, per specimen type, with infection by}\\ \text{pathogen}\times \text{susceptible, I, and resistant to antibiotic y under}\\ \text{surveillance})*100 \end{array}$$ [Eqn 1]

All countries’ results were pulled together, and the median rate of resistance was calculated. Tableau software (Tableau, Mountain View, California, United States) was used to visualise the data though box-and-whisker plots.

The questionnaires’ contents, with countries’ and WHO Regional Offices’ feedback, were pulled together in Microsoft Excel and screened for key words to define codes for the feedback themes. The proportion of respondents for each code was then calculated, based on the total number of questionnaires received.

## Results

### Global Antimicrobial Resistance and Use Surveillance System data calls

#### Countries’ participation and reporting

Enrolment and reporting varied during the three data calls, both on submission on the information of the status of the national AMR surveillance systems and AMR data. By the end of the last data call in August 2019, 23 out of 54 (43%) African countries were formally enrolled in GLASS: Cote D’Ivoire, Egypt, Ethiopia, Gabon, Gambia, Ghana, Kenya, Liberia, Libya, Madagascar, Malawi, Mali, Mauritania, Mauritius, Mozambique, Nigeria, South Africa, Sudan, Tanzania, Tunisia, Uganda, Zambia and Zimbabwe. Table 1 shows progress in country participation and reporting from 2017 to 2019, with almost a 100% increase in number of countries over the three years.

**TABLE 1:** Number of countries on the African continent enrolled in GLASS and which reported information on their national surveillance systems and AMR data during the three GLASS data calls (2017–2019).

Data call (year)	Number of countries on the African continent enrolled in GLASS	Number of countries reporting data to GLASS			
		Information on national surveillance system reported (%)		AMR data (%)	
		*N*	%	*N*	%
2017	11	11	100	6	55
2018	18	18	100	9	50
2019	23	19	83	10	43

GLASS, Global Antimicrobial Resistance and Use Surveillance System; AMR, antimicrobial resistance.

Most countries have been able to report AMR data with age and gender stratification (Table 2). The reporting of the number of tested patients, the denominator used to calculate frequency of AMR infection in patients with suspected bacterial infection, has increased from one (17%) reporting country in 2017, to 10 (100%) reporting countries in 2019. Infection origin has proved to be the least reported variable throughout the three data calls.

**TABLE 2:** Number of countries on the African continent enrolled in GLASS and reporting AST results during the three data GLASS calls (2017–2019), as well as availability of data stratification by age, gender and infection origin and data on the number of patients from which a diagnostic sample was taken (tested).

Data call (year)	Number of countries reporting patients AST results	List of countries	Number of countries reporting additional variables (%)							
			Age		Gender		Infection origin		Number of tested patients	
			*N*	%	*N*	%	*N*	%	*N*	%
2017	6	Egypt, Madagascar, Malawi, South Africa, Tunisia, Zambia	4	67	5	83	4	67	1	17
2018	9	Egypt, Madagascar, Malawi, Nigeria, South Africa, Sudan, Tunisia, Uganda, Zambia	8	89	9	100	4	44	6	67
2019	10	Egypt, Ethiopia, Madagascar, Mali, Mozambique, Nigeria, South Africa, Sudan, Tunisia, Uganda	10	100	10	100	7	70	10	100

GLASS, Global Antimicrobial Resistance and Use Surveillance System; AST, Antimicrobial Susceptibility Testing.

#### Information of the status of national antimicrobial resistance surveillance systems

Data show that the National Coordinating Centre is established, or is in the process of being established, and the National Reference Laboratories are nominated in around 80% of countries participating in the three data calls. The number of surveillance sites reporting to GLASS over the three years went from 52 hospital and five outpatient facilities in 2017, to 63 hospital and 62 outpatient facilities in 2019. In certain cases, the number of surveillance sites could not be retrieved so the number of laboratories supporting the surveillance systems was reported instead; this was done for five laboratories in 2018, and 12 laboratories in 2019. Overall, the total number of surveillance sites increased from 57 in 2017 to 137 in 2019. Almost 80% of countries reported in three data calls having the National Reference Laboratories participating in an External Quality Assessment scheme, while External Quality Assessment for clinical laboratories that contribute to the national AMR surveillance programmes went from being performed by 27% of countries in 2017, to 61% of countries in 2018, and 48% of countries in 2019. In around 70% of the countries, clinical laboratories performed AST according to recognised standards, from either the Clinical & Laboratory Standards Institute or the European Committee on Antimicrobial Susceptibility Testing.

#### Antimicrobial resistance data

**Distribution of infections and bacteria analysed:** Compared to the first data call, with six countries reporting AST results for 11 060 patients with confirmed bacterial infections, in 2019 GLASS received AST results from 10 countries for 32 117 patients, three times the number from 2017 (Table 3). Bloodstream infection is the most frequent infection reported for the three years, followed by UTI, gastroenteric infection and gonorrhoea (Table 3). Bloodstream infections caused by *S. aureus* and *K. pneumoniae* appeared to be the most recurrent, while *E. coli* is the most frequent etiological agent of reported UTIs. Rate of gastroenteric infections caused by *Shigella* species and *Salmonella* species were reported equally through the years.

**TABLE 3:** Summary of confirmed bacterial infections and patient with AST results, by infection site and pathogen, reported by Egypt, Ethiopia, Madagascar, Malawi, Mali, Mozambique, Nigeria, South Africa, Sudan, Tunisia, Uganda, and Zambia during three in GLASS data calls (2017–2019).

Infection site	Pathogen	2017 (*n* = 6)	2018 (*n* = 9)	2019 (*n* = 10)
**Number of patients with confirmed bacterial infection**				
Bloodstream	*Acinetobacter* spp.	141	1307	3264
	*Escherichia coli*	405	1125	4053
	*Klebsiella pneumoniae*	329	481	6146
	*Salmonella* spp.	122	326	616
	*Staphylococcus aureus*	867	1018	6006
	*Streptococcus pneumoniae*	647	896	606
	Total	2511	5153	20 691
Urinary tract	*E. coli*	6731	2351	10 162
	*K. pneumoniae*	1545	1172	2549
	Total	8276	3523	12 711
Gastroenteric	*Salmonella* spp.	83	646	674
	*Shigella* spp.	110	692	807
	Total	193	1338	1481
Genital	*Neisseria gonorrhoeae*	413	938	340
	Total	413	938	340
Total		11 393	10 952	35 223
**Number of patients with AST results**				
Bloodstream	*Acinetobacter* spp.	140	486	2980
	*E. coli*	405	1125	3635
	*K. pneumoniae*	329	481	5540
	*Salmonella* spp.	122	326	568
	*S. aureus*	730	1018	5603
	*S. pneumoniae*	481	896	405
	Total	2207	4332	18 731
Urinary tract	*E. coli*	6706	2300	9343
	*K. pneumoniae*	1541	1161	2315
	Total	8247	3461	11 658
Gastroenteric	*Salmonella* spp.	83	599	629
	*Shigella* spp.	110	650	759
	Total	193	1249	1388
Genital	*N. gonorrhoeae*	413	938	340
	Total	413	938	340
Total		11 060	9980	32 117

GLASS, Global Antimicrobial Resistance and Use Surveillance System; AST, Antimicrobial Susceptibility Testing; *n*, number; spp., species.

**Antimicrobial resistance rates:** The patterns of resistance of *E. coli* and *K. pneumoniae* to different antibiotics by infections sites and specific organisms between 2016 and 2018, as reported in 2017–2019 data calls, are summarised in Table 4. Results show that resistance to 3GC in BSIs is above 50% for *E. coli* and 81% for *K. pneumoniae*, while carbapenem resistance reaches a maximum of 8% for *E. coli* and 24% for *K. pneumoniae*; MRSA is found to be the cause of about 20% of BSIs. Similarly, resistance of *E. coli* and *K. pneumoniae* to carbapenems is generally found to be below 10% in UTIs, while resistance to ciprofloxacin is between 36% and 60%.

**TABLE 4:** Proportion of resistance (median) for selected infection site, by antibiotics and pathogen, in Egypt, Ethiopia, Madagascar, Malawi, Mali, Mozambique, Nigeria, South Africa, Sudan, Tunisia, Uganda and Zambia between 2016 and 2018.

Infection site	Antibiotic	Pathogen	Proportion of resistance (median)		
			2016	2017	2018
Bloodstream infection	Carbapenems	*Escherichia coli*	0.4	7.7	5.0
		*Klebsiella pneumoniae*	23.4	15.2	20.4
	Penicillinase beta lactams	*Staphylococcus aureus*	24.7	22.7	21.1
	Third-generation cephalosporins	*E. coli*	61.9	51.5	50.3
		*K. pneumoniae*	81.8	84.4	84.5
Urinary tract infection	Carbapenems	*E. coli*	0.7	0.7	7.3
		*K. pneumoniae*	17.1	9.7	9.4
	Ciprofloxacin	*E. coli*	57.4	48.2	59.7
		*K. pneumoniae*	58.3	36.3	49.1

**FIGURE 1:**
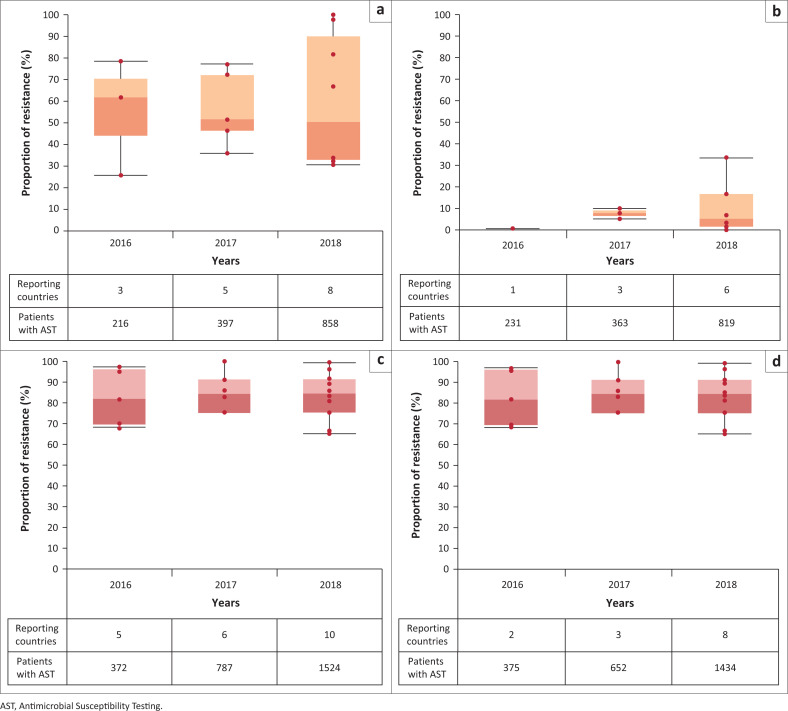

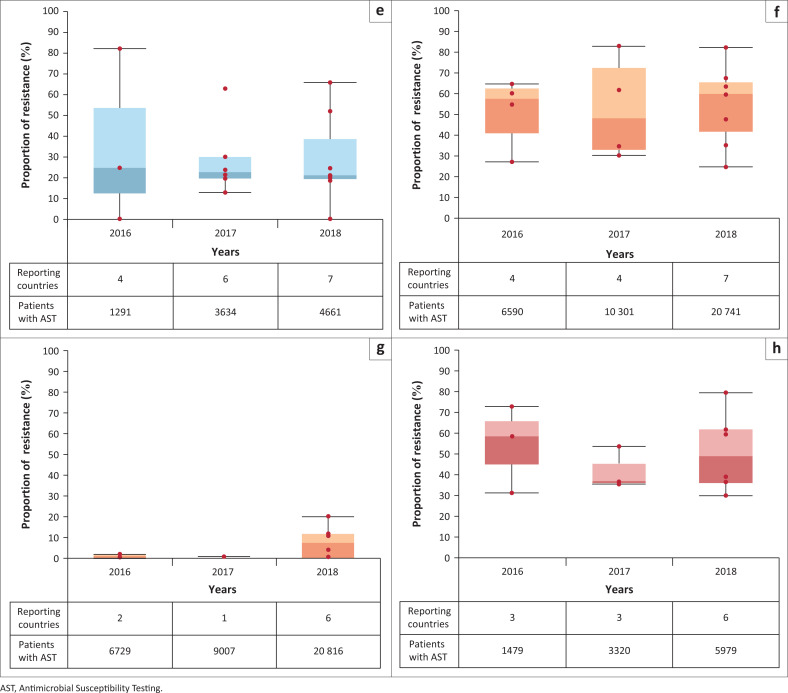

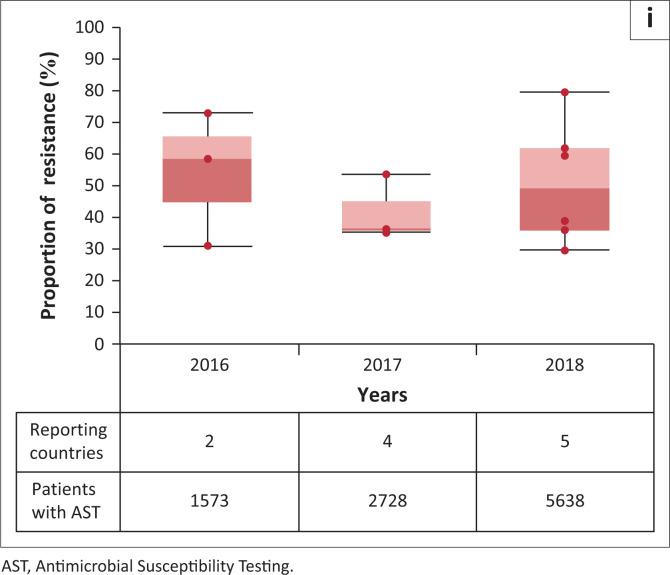
Boxplots showing proportion (median) of infection syndrome due to bacteria resistant to selected antibiotics in Egypt, Ethiopia, Madagascar, Malawi, Mali, Mozambique, Nigeria, South Africa, Sudan, Tunisia, Uganda, and Zambia between 2016 and 2018. The number of countries reporting for each year and the number of patients with Antimicrobial Susceptibility Testing (AST) results are shown in the *x*-axis. The list and the number of countries reporting each year may vary. Each red dot corresponds to a single country result. (a) Boxplots showing proportion (median) of *bloodstream infections due to Escherichia coli resistance to third-generation cephalosporins* in Egypt, Ethiopia, Madagascar, Malawi, Mali, Mozambique, Nigeria, South Africa, Sudan, Tunisia, Uganda and Zambia between 2016 and 2018. The number of countries reporting for each year and the number of patients with AST results are shown below the *x*-axis. The list and the number of countries reporting each year may vary. Each red dot corresponds to a single country result. (b) Boxplots showing proportion (median) of *bloodstream infections due to Escherichia coli resistance to carbapenems* in Egypt, Ethiopia, Madagascar, Malawi, Mali, Mozambique, Nigeria, South Africa, Sudan, Tunisia, Uganda and Zambia between 2016 and 2018. The number of countries reporting for each year and the number of patients with AST results are shown below the *x*-axis. The list and the number of countries reporting each year may vary. Each red dot corresponds to a single country result. (c) Boxplots showing proportion (median) *of bloodstream infections due to Klebsiella pneumoniae resistance to third-generation cephalosporins*, in Egypt, Ethiopia, Madagascar, Malawi, Mali, Mozambique, Nigeria, South Africa, Sudan, Tunisia, Uganda and Zambia, between 2016 and 2019. The number of countries reporting for each year and the number of patients with AST results are shown below the *x*-axis. The list and the number of countries reporting each year may vary. Each red dot corresponds to a single country result. (d) Boxplots showing proportion (median) of *bloodstream infections due to Klebsiella pneumoniae resistance to carbapenes* in Egypt, Ethiopia, Madagascar, Malawi, Mali, Mozambique, Nigeria, South Africa, Sudan, Tunisia, Uganda and Zambia, between 2016 and 2019. The number of countries reporting for each year and the number of patients with AST results are shown below the *x*-axis. The list and the number of countries reporting each year may vary. Each red dot corresponds to a single country result. (e) Boxplots showing proportion (median) of *bloodstream infections due to methicillin-resistant* *Staphylococcus aureus* (MRSA) in Egypt, Ethiopia, Madagascar, Malawi, Mali, Mozambique, Nigeria, South Africa, Sudan, Tunisia, Uganda and Zambia, between 2016 and 2019. The number of countries reporting for each year and the number of patients with AST results are shown below the *x*-axis. The list and the number of countries reporting each year may vary. Each red dot corresponds to a single country result. (f) Boxplots showing proportion (median) *of urinary tract infections due to Escherichia coli resistance to ciprofoloxacin* in Egypt, Ethiopia, Madagascar, Malawi, Mali, Mozambique, Nigeria, South Africa, Sudan, Tunisia, Uganda and Zambia between 2016 and 2019. The number of countries reporting for each year and the number of patients with AST results are shown below the *x*-axis. The list and the number of countries reporting each year may vary. Each red dot corresponds to a single country result. (g) Boxplots showing proportion (median) *of urinary tract infections due to Escherichia coli* resistance to carbapenems in Egypt, Ethiopia, Madagascar, Malawi, Mali, Mozambique, Nigeria, South Africa, Sudan, Tunisia, Uganda and Zambia between 2016 and 2019. The number of countries reporting for each year and the number of patients with AST results are shown below the *x*-axis. The list and the number of countries reporting each year may vary. Each red dot corresponds to a single country result. (h) Boxplots showing proportion (median) *of urinary tract infections due to Klebsiella pneumoniae resistance to ciprofloxacin* in Egypt, Ethiopia, Madagascar, Malawi, Mali, Mozambique, Nigeria, South Africa, Sudan, Tunisia, Uganda and Zambia between 2016 and 2019. The number of countries reporting for each year and the number of patients with AST results are shown below the *x*-axis. The list and the number of countries reporting each year may vary. Each red dot corresponds to a single country result. (i) Boxplots showing proportion (median) of *urinary tract infections due to Klebsiella pneumoniae resistance to carbapenems* in Egypt, Ethiopia, Madagascar, Malawi, Mali, Mozambique, Nigeria, South Africa, Sudan, Tunisia, Uganda and Zambia between 2016 and 2019. The number of countries reporting for each year and the number of patients with AST results are shown below the *x*-axis. The list and the number of countries reporting each year may vary. Each red dot corresponds to a single country result.

Boxplots for each pathogen–antibiotic combination in different infections sites (BSI and UTI) are presented in Figure 1. Although the list and number of countries reporting on specific pathogen–antibiotic combination varied throughout the three data calls, data show a certain consistency in the reported rates.

### Countries’ and WHO Regional Offices’ feedback

**FIGURE 2:**
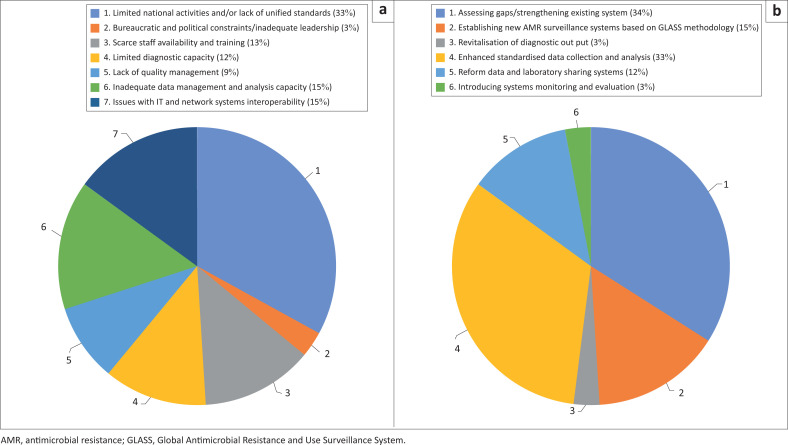

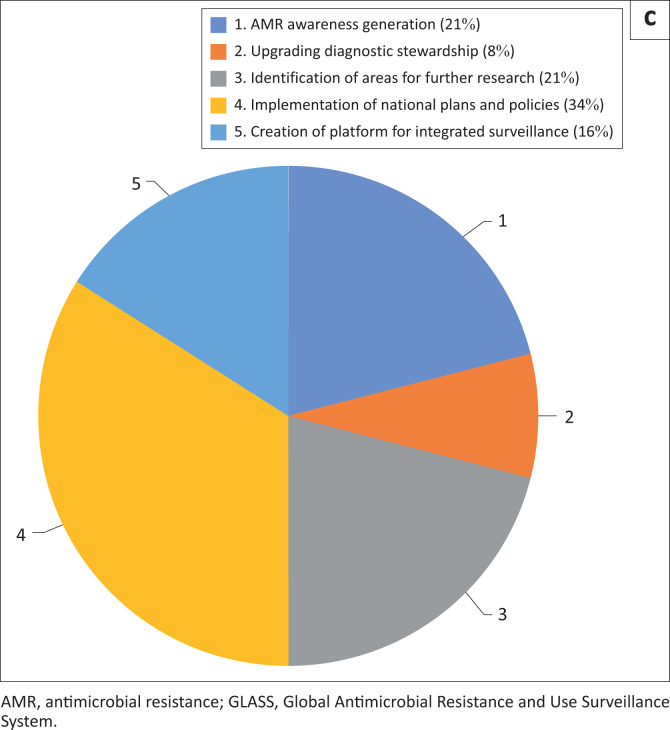
Pie charts summarising the proportion of countries’ National Focal Points and WHO Regional Offices’ responses related to constraints, perceived impact, and value associated to reporting data to Global Antimicrobial Resistance and Use Surveillance System (GLASS). Feedback was received by Kenya, Madagascar, Malawi, Nigeria, South Africa, Sudan, Tanzania, Tunisia, Uganda, Zambia, Zimbabwe and the WHO Regional Office for Africa and Regional Office for the East Mediterranean, in relation to 2017 and 2019 GLASS data calls. (a) Pie chart summarising the proportion of countries’ National Focal Points and WHO Regional Offices’ responses related to constraints associated to reporting data to GLASS. Feedback was received by Kenya, Madagascar, Malawi, Nigeria, South Africa, Sudan, Tanzania, Tunisia, Uganda, Zambia, Zimbabwe and the WHO Regional Office for Africa and Regional Office for the East Mediterranean, in relation to 2017 and 2019 GLASS data calls. (b) Pie chart summarising the proportion of countries’ National Focal Points and WHO Regional Offices’ responses related to perceived impact associated to reporting data to GLASS. Feedback was received by Kenya, Madagascar, Malawi, Nigeria, South Africa, Sudan, Tanzania, Tunisia, Uganda, Zambia, Zimbabwe and the WHO Regional Office for Africa and Regional Office for the East Mediterranean, in relation to 2017 and 2019 GLASS data calls. (c) Pie chart summarising the proportion of countries’ National Focal Points and WHO Regional Offices’ responses related to perceived value associated to reporting data to GLASS. Feedback was received by Kenya, Madagascar, Malawi, Nigeria, South Africa, Sudan, Tanzania, Tunisia, Uganda, Zambia, Zimbabwe and the WHO Regional Office for Africa and Regional Office for the East Mediterranean, in relation to 2017 and 2019 GLASS data calls.

Eleven countries, as well as the WHO Regional Office for Africa and the WHO Regional Office for the Eastern Mediterranean, provided feedback on the first three years of GLASS implementation. The feedback from NFPs was provided either at the end of the first data call, the third data call, or both (Table 5). Responses by NFPs and Regional Offices are presented in Figure 2 by the three themes (constraint, perceived impact, and value) and identified codes. Limited resources, economic issues, bureaucratic bottlenecks, and political instability were reported as having a major impact on the roll out of national AMR surveillance systems. For example, the partial absence of quality assurance provision and tools to extract, clean and aggregate data were listed as important limitations to countries’ participation. Scarce availability of trained professionals and resources to hire new staff was also indicated as major issue. Both WHO Regional Offices noted that GLASS data generation, validation, and processing are centralised and headed by a single NFP. However, due to the shortage of manpower, NFPs frequently oversee other activities, which results in competing priorities that might delay data reporting. Lack of IT tools, technical training for data management and data preparation, and complex IT system interoperability, were also mentioned as important constraint for data reporting. In some countries, where a national laboratory-based surveillance programme has been in place for a long period of time, an enhanced surveillance system had to be adapted to meet GLASS’s capacity to include population data.

**TABLE 5:** List of countries and years for which countries National Focal Points provided feedback to GLASS.

Countries	National Focal Points feedback	
	1st GLASS data call (2017)	3rd GLASS data call (2019)
Kenya	-	x
Madagascar	x	x
Malawi	x	-
Nigeria	x	-
South Africa	x	x
Sudan	-	x
Tanzania	-	x
Tunisia	x	-
Uganda	x	x
Zambia	x	x
Zimbabwe	x	-

Note: Feedback was received by Kenya, Madagascar, Malawi, Nigeria, South Africa, Sudan, Tanzania, Tunisia, Uganda, Zambia, Zimbabwe in relation to 2017 and 2019 GLASS data calls.

GLASS, Global Antimicrobial Resistance and Use Surveillance System.

Nevertheless, the GLASS surveillance methods proposed, and the IT tools offered, were found to be well-defined and easy to use. Countries were able to reform data sharing systems to suit the GLASS data reporting model. Mostly, this was done by establishing local laboratories data systems, which were electronically linked to clinical data and to national network. According to one NFP, feedback was also sent to hospitals and outpatient clinics, resulting in treatment changes. The application of WHONET, a laboratory information system software, to GLASS data preparation was found to be very useful and succeeded to strengthen data management capability.^12^ The national reports produced using data collected for GLASS were used by countries to develop and/or implement AMR national plans and policies, to develop proposals, to orient partners, and to direct the necessary technical assistance. Participation in GLASS has helped to launch strategies on data use and development of policies on antimicrobial use and AMR. The experience gained during the data calls has been used to establish the mechanism of monitoring and evaluation of the AMR surveillance system. The evidence has also collectively brought together One Health partners and ministries to implement multisectoral projects and integrated surveillance.

## Discussion

African countries have responded to the first GLASS three data calls with a high level of interest and dedication, and the GLASS framework has proven to be a vital tool to the establishment and/or development of national AMR surveillance systems, as reflected by the overall positive feedback of NFPs to GLASS participation. The main constraints encountered by countries during the data calls were linked to lack of specific national activities to tackle AMR, scarce laboratory capability, staffing and budget issues, and data management. However, through support from GLASS, countries were also able to revitalise their laboratory components and microbiological output, both for infectious diseases and AMR diagnostics.

In order to respond to the GLASS data call, countries improved the collection, analysis and presentation of standardised data generated from healthcare facilities, which in some cases also resulted in improved patient treatment. Countries without an AMR surveillance system in place used the GLASS Manual for Early Implementation to model the structure of the new national system.^13^ The initial steps of the participation and the data call also pushed countries to assess the capacity of their national AMR reporting system(s). This allowed the identification of gaps to address in the future, and it acted in participating countries as platform for the establishment of the national surveillance core components.

This is key, considering that the data reported to all three GLASS data calls (2017–2019) might suggest the presence of high rates of AMR in the continent. As expected, AST results for BSI were most frequently reported, followed by UTI, gastroenteric infection and gonorrhoea. This is in line with the available evidence which shows that, in Africa, BSI is a major cause of morbidity and mortality, and UTIs are some of the most frequent bacterial infections affecting people, both in the community and in hospitals.^7^

Reported high resistance to 3GC is particularly worrying in some parts of Africa, where diagnostic facilities are scarce and antibiotics such as carbapenems and semi-synthetic aminoglycosides (e.g. amikacin) are either unavailable or prohibitively expensive.^7^ In many sub-Saharan Africa hospitals, limited nursing capacity favours the use of broad-spectrum antimicrobials with a once-daily dosing regimen and this has led to the widespread adoption of 3GC for the empirical management of hospitalised patients with suspected sepsis.^14^ Moreover, extended spectrum beta-lactamase-producing *Enterobacterales*, for which resistance to 3GC is a marker, are also resistant to penicillins and therefore represent an important threat to the treatment of BSIs in these settings.^7^

The reported rate of BSI caused by MRSA was also high (between 21% and 24%). Methicillin-resistant *S. aureus* has been linked to significant morbidity and mortality and it carries an evident threat to African countries, since there might be limited access to antibiotics effective against hospital-associated MRSA, such as linezolid and daptomycin.^15^ Furthermore, the scarce implementation of infection prevention and control measures and widespread HIV infection and tuberculosis can amplify the difficulty of dealing with the MRSA epidemic in Africa.

*Escherichia coli*- and *K. pneumoniae*-reported resistance to ciprofloxacin in UTIs was found to be consistently high (between 36% and 60%). This could potentially be linked to samples obtained from complicated and hospitalised patients, as in almost all countries reporting to the GLASS community, UTIs are not tested for and treated empirically.^3,16^ This is important, as fluoroquinolones have an significant role in treating of severe infections, such as septicaemia, and therefore increasing resistance can have severe health consequences.^17^ Finally, reported resistance of *E. coli* and *K. pneumoniae* to carbapenems was high in both BSIs and UTIs. This is worrying as, until recently, carbapenems were the last-resort antibiotics used for managing multidrug-resistant bacterial infections.^18^ Moreover, the organisms that are resistant to carbapenems are frequently resistant to many other classes of commonly-used antimicrobial agents; thus, managing infections caused by them poses a substantial challenge in clinical practice and their public health impacts cannot be over-emphasised.^19,20^

Both ciprofloxacin and carbapenems are on the ‘watch’ list of the WHO 2019 AWaRe Classification, that comprises antibiotics with higher potential to induce resistance; ciprofloxacin is also on the WHO Model List of Essential Medicines, where it is listed as a first- or second-choice empirical treatment option for definite infectious syndromes.^21^ Considering the reported AMR data, the spread of all listed resistant patterns needs to be carefully monitored, and every country should apply measures for continuous data collection, by strengthening surveillance activities or implementing population-based studies (e.g. prevalence survey).

### Limitations

Due to the quality of the data reported to GLASS, and associated potential bias, no trends analysis was performed with presented AMR data, nor comparisons among infection types, or the identification of risk factors linked to age, gender or the source of infection. As stated in the GLASS report 2020^8^:

[*D*]ata aggregation is a major limitation, as it considerably limits options for epidemiological characterization, obviating the detection and validation of data from countries … with unusual antimicrobial patterns. Furthermore, … [l]ack of a sampling strategy results in selection bias, which may affect the representativeness and precision of results. Cases are found and tested only in the population that seeks medical care, … and most data are still generated in laboratories, with no epidemiological insight.

Antimicrobial susceptibility testing varied widely among countries for the specimen–pathogen–antibiotic combinations chosen. The numbers of patients screened for resistance were still very low, suggesting that most data come from complicated and hospitalised patients. Unfortunately, it was also not possible to show the frequency of AMR for these syndrome-pathogen–antibiotic combinations in the tested population, as the needed denominator – the population of the patient for which a diagnostic sample is taken – was not always available.^8^ Finally, it was not possible to obtain feedback from all of the African countries participating in the GLASS data calls. However, responses showed a homogeneous consensus to the global system participation and the benefits associated to it.

### Conclusion

Although some African countries listed in this article still face important constraints while building their national AMR surveillance systems, and even if not all of them have provided AMR data, countries have shown a willingness to share information with GLASS, particularly the status and the development of their surveillance systems. Countries on the continent are working towards reaching a status that will enable them to report data in a complete and systematic manner, through the establishment of surveillance core components and by assuring the quality of AMR diagnostics. Although reported data are still not representative at a national level, and AST varied considerably among countries, the participation in GLASS is clearly linked to improved national surveillance systems, which will also result in better clinical care in prescribing the appropriate antibiotics, one of the most challenging objectives of the Global Action Plan-AMR.

Future improvements involve the expansion of routine surveillance capacity for several countries and the implementation of surveys that allow for effective definition of the magnitude of AMR on the continent. Meanwhile, the evidence generated is supporting the identification of areas for further research – AMR burden in healthcare settings, improvement of diagnostic stewardship, and AMR in the human animal environment interface – and it is advocating for the continuous support of actions directed to AMR monitoring and control.

Together with both national and regional partners, African countries’ participation in GLASS is leading the way towards the further development of an efficient and reliable global surveillance system, which will be able to function in various economic and socio-political contexts, and provide vital and actionable data. Addressing antimicrobial resistance through GLASS is part of the ongoing efforts of Member States to strengthen health security, improve health systems and ensure Universal Health Coverage.^22^
